# Crystal structure of aqua­(1*H*-pyrazole-κ*N* ^2^)(pyridine-2,6-di­carboxyl­ato-κ^3^ *O* ^2^,*N*,*O* ^6^)copper(II) dihydrate

**DOI:** 10.1107/S2056989017016231

**Published:** 2017-11-17

**Authors:** Daeyoung Kim, Sung Kwon Kang

**Affiliations:** a Department of Chemistry, Chungnam National University, Daejeon 34134, Republic of Korea

**Keywords:** crystal structure, pyridine-2,6-di­carboxyl­ate, pyrazole, square-pyramidal Cu^II^ complex

## Abstract

In the title compound, [Cu(C_7_H_3_NO_4_)(C_3_H_4_N_2_)(H_2_O)]·2H_2_O, the Cu^II^ atom is coordinated by three O atoms and two N atoms, provided by a tridentate pyridine-2,6-di­carboxyl­ate (pdc), one pyrazole and one water ligand, forming a slightly distorted square-pyramidal geometry [range of O—Cu—O and O—Cu—N bond angles = 79.55 (8)–166.22 (10)°]. The water mol­ecule is positioned at the apical position. In the crystal, the complex mol­ecule and the two crystallographically independent non-coordinating water mol­ecules are linked into a supra­molecular layer structure parallel to the *ab* plane *via* O—H⋯O and N—H⋯O hydrogen bonds.

## Abstract 2

The synthesis and crystal structure of tridentate pyridine-2,6-di­carboxyl­ate Cu^II^ complex with a heterocyclic pyrazole ligand, a potential candidate for metal catalysts, are reported. The Cu^II^ atom is coordinated by three O atoms and two N atoms, provided by a tridentate pyridine-2,6-di­carboxyl­ate, one pyrazole and one water ligand, forming a slightly distorted square-pyramidal geometry.

## Chemical context   

Metal complexes with the tridentate ligand 2,6-bis­[(1*H*-pyrazol-1-yl)meth­yl]pyridine are known to be catalysts of polyethyl­ene polymerization (Singh *et al.*, 2003 ▸; Watson *et al.*, 1987 ▸; Son *et al.*, 2014 ▸; Kim & Kang, 2015 ▸). 2,6-Bis[(1*H*-pyrazol-1-yl)meth­yl]pyridine was oxidized to pyridine-2,6-di­carboxyl­ate (pdc) by metal nitrate (Unuigboje & Anyile, 2007 ▸). The pdc mol­ecule has been recognized as a component of bacterial spores, and is also useful in a variety of processes as an enzyme inhibitor, plant preservative and food sanitizer (Cui *et al.*, 2011 ▸). The pdc mol­ecule has been selected as a primary dibasic tridentate ligand and a metal complex with pdc was reported to be a new chemical sensor (Mistri *et al.*, 2013 ▸). Attention has been paid to the design of various *N*-donor ligands with special structural properties in order to investigate the specific stereochemical requirements of a particular metal-binding site (Mukherjee, 2000 ▸). Various substituted *N*-donor heterocyclic ligands such as imidazole and pyrazole have been selected as a second ligand, so that the structural and electronic effects on the biologically important Cu—N bond could be probed (Ang *et al.*, 1991 ▸; Chen *et al.*, 2011 ▸; Lin *et al.*, 2009 ▸; Liu *et al.*, 2005 ▸). As part of these continuing studies, the title complex has been synthesized and characterized by single crystal X-ray diffraction.

## Structural commentary   

**Figure 1:**
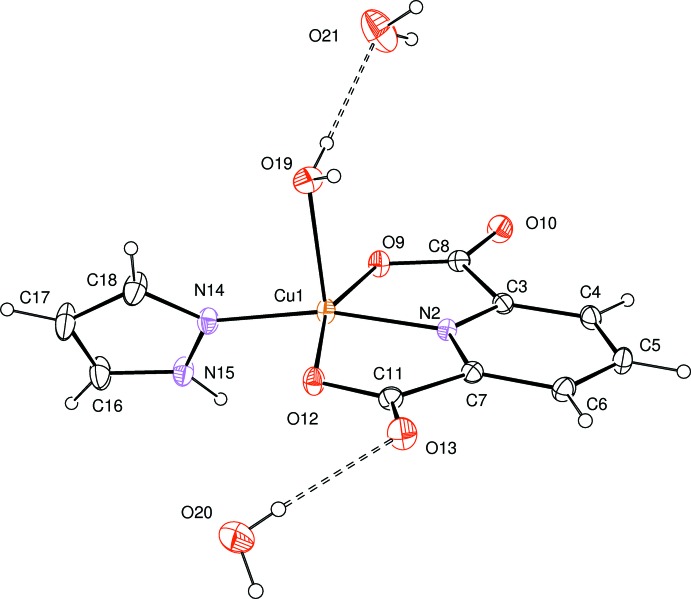
The mol­ecular structure of the title compound, showing the atom-numbering scheme and 30% probability ellipsoids for non-H atoms. H atoms are drawn as small spheres of arbitrary radii. The O—H⋯O hydrogen bonds are indicated by dashed lines.

The mol­ecular structure of the title compound is shown in Fig. 1 ▸. The Cu^II^ atom is coordinated by three O atoms and two N atoms from tridentate pyridine-2,6-di­carboxyl­ate (pdc), pyrazole and water ligands. The coordination geometry around the Cu^II^ atom is a distorted square pyramid as indicated by the τ value of 0.113 (Addison *et al.*, 1984 ▸). The Cu^II^ atom lies in the center of the basal plane defined by two nitro­gen atoms (N2 from pdc and N14 from pyrazole) and two oxygen atoms (O9 and O12 from pdc). The plane including the Cu^II^ atom is almost planar, with an r.m.s. deviation of 0.0847 Å from the corresponding least-squares plane defined by the five constituent atoms. The pyrazole ring is twisted by 66.61 (10)° from the basal plane. The apical Cu1—O19 bond length of 2.217 (2) Å is much longer than those of the basal Cu—O lengths [Cu1—O9 = 2.026 (2) Å and Cu1—O12 = 2.058 (2) Å].
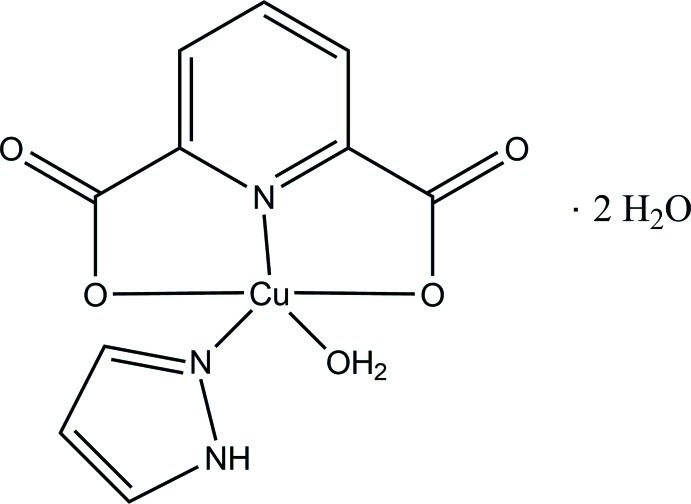


## Supra­molecular features   

**Figure 2:**
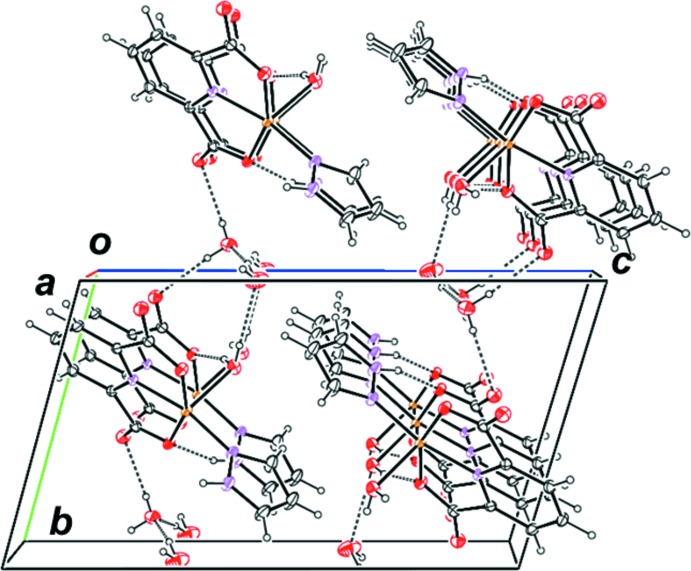
Part of the packing diagram of the title compound, showing mol­ecules linked by inter­molecular O—H⋯O and N—H⋯O hydrogen bonds (dashed lines).

In the crystal, O—H⋯O hydrogen bonds (O19—H19*B*⋯O21, O20—H20*B*⋯O13 and O20—H20*A*⋯O10^iii^; symmetry code as in Table 1 ▸) link the complex mol­ecule to the non-coordinating water mol­ecules (Fig. 1 ▸). Two crystallographically independent non-coordinating water mol­ecules are also linked to each other by O—H⋯O hydrogen bonds (O21—H21*A*⋯O20^iv^ and O21—H21*B*⋯O20^v^; Table 1 ▸). Adjacent complex mol­ecules are connected by other O—H⋯O and N—H⋯O hydrogen bonds (N15—H15⋯O12^i^ and O19—H19*A*⋯O9^ii^; Table 1 ▸). The above-mentioned inter­molecular inter­actions stabilize and link the components into a two-dimensional network parallel to the *ab* plane (Fig. 2 ▸).

## Database survey   

A search of the Cambridge Structural Database (Version 5.37 with two updates, Groom *et al.*, 2016 ▸) returned 1448 entries for crystal structures related to the name pyridine-2,6-di­carboxyl­ato. Most of them are crystal structures of metal complexes. However, there are only four entries with a secondary ligand of a pyrazolyl derivative bonded to a transition metal, *viz*. a Cu complex (Lin *et al.*, 2009 ▸; Wang *et al.*, 2014 ▸) and Zn and Co complexes (Zhang *et al.*, 2011 ▸).

## Synthesis and crystallization   

A solution of copper nitrate trihydrate (0.072 g, 0.3 mmol) in aceto­nitrile (5 ml) was added to a solution of 2,6-bis­[(1*H*-pyra­zol-1-yl)meth­yl]pyridine (0.072 g, 0.3 mmol) in aceto­nitrile (5 ml) in a high-pressure vessel. After sealing the high-pressure vessel, the resulting solution was stirred for three days at 403 K. The precipitate formed was removed by filtration, and the filtrate was washed with aceto­nitrile and di­chloro­methane to get a dark-green powder. Single crystals of the title compound were obtained from its aqueous solution by slow evaporation of the solvent at 333 K within five days.

## Refinement   

Crystal data, data collection and structure refinement details are summarized in Table 2 ▸. H atoms of the water mol­ecules and the NH group were located in a difference-Fourier map and refined freely [refined distances; O—H = 0.70 (5)–0.91 (6) Å and N—H = 0.93 (4) Å]. Other H atoms were positioned geometrically and refined using a riding model, with C—H = 0.93 Å, and with *U*_iso_(H) = 1.2*U*_eq_(C).

## Supplementary Material

Crystal structure: contains datablock(s) I. DOI: 10.1107/S2056989017016231/is5480sup1.cif

Structure factors: contains datablock(s) I. DOI: 10.1107/S2056989017016231/is5480Isup2.hkl

CCDC reference: 1584872

Additional supporting information:  crystallographic information; 3D view; checkCIF report

## Figures and Tables

**Table 1:** Hydrogen-bond geometry (Å, °)

*D*—H⋯*A*	*D*—H	H⋯*A*	*D*⋯*A*	*D*—H⋯*A*
O19—H19*B*⋯O21	0.75 (4)	2.09 (5)	2.831 (5)	169 (4)
O20—H20*B*⋯O13	0.70 (5)	2.12 (5)	2.807 (4)	172 (6)
N15—H15⋯O12^i^	0.93 (4)	1.93 (4)	2.832 (3)	164 (4)
O19—H19*A*⋯O9^ii^	0.70 (5)	2.12 (5)	2.805 (3)	165 (5)
O20—H20*A*⋯O10^iii^	0.78 (5)	2.01 (5)	2.784 (4)	171 (5)
O21—H21*A*⋯O20^iv^	0.91 (6)	2.05 (6)	2.933 (5)	163 (5)
O21—H21*B*⋯O20^v^	0.81 (6)	2.17 (6)	2.938 (5)	161 (6)

Symmetry codes: (i) 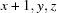; (ii) 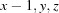; (iii) 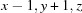; (iv) 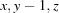; (v) 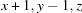.

**Table 2:** Experimental details

Crystal data	
Chemical formula	[Cu(C_7_H_3_NO_4_)(C_3_H_4_N_2_)(H_2_O)]·2H_2_O
*M*_r_	350.77
Crystal system, space group	Triclinic, *P* 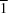
Temperature (K)	296
*a*, *b*, *c* (Å)	5.2171 (9), 8.9249 (16), 15.309 (3)
α, β, γ (°)	105.289 (8), 94.523 (8), 93.295 (9)
*V* (Å^3^)	683.2 (2)
*Z*	2
Radiation type	Mo *K*α
μ (mm^−1^)	1.64
Crystal size (mm)	0.25 × 0.23 × 0.12

Data collection	
Diffractometer	Bruker SMART CCD area-detector
Absorption correction	Multi-scan (*SADABS*; Bruker, 2012 ▸)
*T*_min_, *T*_max_	0.546, 0.726
No. of measured, independent and observed [*I* > 2σ(*I*)] reflections	15587, 3312, 3110
*R*_int_	0.024
(sin θ/λ)_max_ (Å^−1^)	0.667

Refinement	
*R*[*F* ^2^ > 2σ(*F* ^2^)], *wR*(*F* ^2^), *S*	0.035, 0.088, 1.15
No. of reflections	3312
No. of parameters	214
H-atom treatment	H atoms treated by a mixture of independent and constrained refinement
Δρ_max_, Δρ_min_ (e Å^−3^)	0.68, −0.53

Computer programs: *SMART* and *SAINT* (Bruker, 2012 ▸), *SHELXS2013* (Sheldrick, 2008 ▸), *SHELXL2013* (Sheldrick, 2015 ▸) and *ORTEP-3 for Windows* and *WinGX* (Farrugia, 2012 ▸).

## supplementary crystallographic information

### Crystal data

**Table:**

[Cu(C_7_H_3_NO_4_)(C_3_H_4_N_2_)(H_2_O)]·2H_2_O	*Z* = 2
*M**_r_* = 350.77	*F*(000) = 358
Triclinic, *P*1	*D*_x_ = 1.705 Mg m^−^^3^
*a* = 5.2171 (9) Å	Mo *K*α radiation, λ = 0.71073 Å
*b* = 8.9249 (16) Å	Cell parameters from 9694 reflections
*c* = 15.309 (3) Å	θ = 2.4–28.2°
α = 105.289 (8)°	µ = 1.64 mm^−^^1^
β = 94.523 (8)°	*T* = 296 K
γ = 93.295 (9)°	Block, green
*V* = 683.2 (2) Å^3^	0.25 × 0.23 × 0.12 mm

### Data collection

**Table:**

Bruker SMART CCD area-detector diffractometer	3110 reflections with *I* > 2σ(*I*)
Radiation source: fine-focus sealed tube	*R*_int_ = 0.024
φ and ω scans	θ_max_ = 28.3°, θ_min_ = 2.4°
Absorption correction: multi-scan (SADABS; Bruker, 2012)	*h* = −6→6
*T*_min_ = 0.546, *T*_max_ = 0.726	*k* = −11→11
15587 measured reflections	*l* = −20→20
3312 independent reflections	

### Refinement

**Table:**

Refinement on *F*^2^	0 restraints
Least-squares matrix: full	Hydrogen site location: mixed
*R*[*F*^2^ > 2σ(*F*^2^)] = 0.035	H atoms treated by a mixture of independent and constrained refinement
*wR*(*F*^2^) = 0.088	*w* = 1/[σ^2^(*F*_o_^2^) + (0.0181*P*)^2^ + 1.271*P*] where *P* = (*F*_o_^2^ + 2*F*_c_^2^)/3
*S* = 1.15	(Δ/σ)_max_ < 0.001
3312 reflections	Δρ_max_ = 0.68 e Å^−^^3^
214 parameters	Δρ_min_ = −0.52 e Å^−^^3^

### Special details

**Table:**

Geometry. All esds (except the esd in the dihedral angle between two l.s. planes) are estimated using the full covariance matrix. The cell esds are taken into account individually in the estimation of esds in distances, angles and torsion angles; correlations between esds in cell parameters are only used when they are defined by crystal symmetry. An approximate (isotropic) treatment of cell esds is used for estimating esds involving l.s. planes.

### Fractional atomic coordinates and isotropic or equivalent isotropic displacement parameters (Å^2^)

**Table:**

	*x*	*y*	*z*	*U*_iso_*/*U*_eq_	
Cu1	0.72303 (6)	0.46457 (4)	0.26827 (2)	0.02692 (10)	
N2	0.6036 (4)	0.3633 (2)	0.14415 (14)	0.0245 (4)	
C3	0.7285 (5)	0.2455 (3)	0.09966 (17)	0.0262 (5)	
C4	0.6564 (6)	0.1715 (3)	0.00900 (19)	0.0332 (6)	
H4	0.7444	0.0895	−0.0224	0.040*	
C5	0.4473 (6)	0.2238 (3)	−0.03373 (19)	0.0363 (6)	
H5	0.3942	0.1762	−0.0947	0.044*	
C6	0.3182 (6)	0.3459 (3)	0.01379 (19)	0.0328 (6)	
H6	0.1778	0.3807	−0.0143	0.039*	
C7	0.4036 (5)	0.4151 (3)	0.10435 (17)	0.0253 (5)	
C8	0.9466 (5)	0.2086 (3)	0.16040 (18)	0.0288 (5)	
O9	0.9876 (4)	0.3056 (2)	0.23975 (13)	0.0327 (4)	
O10	1.0648 (5)	0.0935 (3)	0.13212 (15)	0.0443 (5)	
C11	0.2971 (5)	0.5503 (3)	0.16952 (18)	0.0282 (5)	
O12	0.4307 (4)	0.6002 (2)	0.24738 (13)	0.0319 (4)	
O13	0.0961 (4)	0.6013 (3)	0.14620 (15)	0.0417 (5)	
N14	0.8880 (4)	0.6023 (3)	0.38160 (16)	0.0330 (5)	
N15	1.1120 (5)	0.6903 (3)	0.39067 (18)	0.0409 (6)	
H15	1.218 (8)	0.680 (5)	0.344 (3)	0.066 (12)*	
C16	1.1553 (7)	0.7888 (4)	0.4734 (2)	0.0530 (9)	
H16	1.2963	0.8616	0.4944	0.064*	
C17	0.9590 (8)	0.7642 (5)	0.5214 (2)	0.0583 (10)	
H17	0.9378	0.8150	0.5814	0.070*	
C18	0.7951 (7)	0.6469 (4)	0.4620 (2)	0.0492 (8)	
H18	0.6417	0.6053	0.4765	0.059*	
O19	0.4765 (5)	0.3250 (3)	0.33442 (17)	0.0397 (5)	
H19A	0.356 (9)	0.304 (5)	0.309 (3)	0.061 (15)*	
H19B	0.521 (8)	0.246 (5)	0.333 (3)	0.057 (14)*	
O20	0.0431 (7)	0.9056 (3)	0.2509 (2)	0.0657 (8)	
H20A	0.061 (10)	0.952 (6)	0.215 (4)	0.079*	
H20B	0.042 (10)	0.828 (6)	0.226 (4)	0.079*	
O21	0.5720 (7)	0.0147 (4)	0.3314 (3)	0.0839 (11)	
H21A	0.420 (11)	−0.039 (7)	0.303 (4)	0.101*	
H21B	0.676 (12)	−0.028 (7)	0.299 (4)	0.101*	

### Atomic displacement parameters (Å^2^)

**Table:**

	*U*^11^	*U*^22^	*U*^33^	*U*^12^	*U*^13^	*U*^23^
Cu1	0.02439 (16)	0.03062 (17)	0.02206 (16)	0.00556 (12)	−0.00065 (11)	0.00077 (12)
N2	0.0246 (10)	0.0259 (10)	0.0219 (10)	0.0018 (8)	0.0023 (8)	0.0047 (8)
C3	0.0272 (12)	0.0239 (11)	0.0268 (12)	0.0010 (9)	0.0047 (10)	0.0053 (9)
C4	0.0398 (15)	0.0276 (13)	0.0290 (13)	0.0014 (11)	0.0078 (11)	0.0011 (10)
C5	0.0445 (16)	0.0381 (15)	0.0217 (12)	−0.0033 (12)	−0.0001 (11)	0.0023 (11)
C6	0.0340 (14)	0.0364 (14)	0.0279 (13)	−0.0002 (11)	−0.0028 (11)	0.0107 (11)
C7	0.0239 (12)	0.0269 (12)	0.0253 (12)	0.0010 (9)	0.0025 (9)	0.0075 (9)
C8	0.0277 (13)	0.0292 (13)	0.0306 (13)	0.0043 (10)	0.0054 (10)	0.0089 (10)
O9	0.0276 (9)	0.0372 (10)	0.0306 (10)	0.0083 (8)	−0.0014 (7)	0.0042 (8)
O10	0.0511 (13)	0.0405 (12)	0.0425 (12)	0.0220 (10)	0.0085 (10)	0.0083 (9)
C11	0.0279 (13)	0.0290 (12)	0.0294 (13)	0.0057 (10)	0.0036 (10)	0.0097 (10)
O12	0.0311 (10)	0.0336 (10)	0.0277 (9)	0.0107 (8)	0.0012 (7)	0.0012 (8)
O13	0.0366 (11)	0.0424 (12)	0.0448 (12)	0.0166 (9)	−0.0044 (9)	0.0089 (9)
N14	0.0282 (11)	0.0387 (13)	0.0280 (11)	−0.0003 (9)	0.0003 (9)	0.0029 (10)
N15	0.0372 (14)	0.0487 (15)	0.0299 (13)	−0.0047 (11)	0.0072 (10)	−0.0011 (11)
C16	0.053 (2)	0.054 (2)	0.0371 (17)	−0.0137 (16)	0.0027 (15)	−0.0095 (15)
C17	0.059 (2)	0.072 (2)	0.0294 (16)	−0.0077 (19)	0.0098 (15)	−0.0112 (16)
C18	0.0427 (18)	0.068 (2)	0.0308 (15)	−0.0053 (16)	0.0080 (13)	0.0026 (15)
O19	0.0297 (12)	0.0496 (14)	0.0428 (13)	0.0017 (10)	0.0000 (10)	0.0188 (11)
O20	0.096 (2)	0.0400 (14)	0.0618 (19)	0.0135 (16)	0.0016 (16)	0.0158 (13)
O21	0.070 (2)	0.0589 (19)	0.106 (3)	0.0035 (16)	−0.021 (2)	0.0010 (18)

### Geometric parameters (Å, º)

**Table:**

Cu1—N2	1.913 (2)	C11—O13	1.231 (3)
Cu1—N14	1.944 (2)	C11—O12	1.288 (3)
Cu1—O9	2.0255 (19)	N14—C18	1.329 (4)
Cu1—O12	2.0577 (19)	N14—N15	1.347 (3)
Cu1—O19	2.217 (2)	N15—C16	1.331 (4)
N2—C3	1.328 (3)	N15—H15	0.93 (4)
N2—C7	1.333 (3)	C16—C17	1.346 (5)
C3—C4	1.382 (4)	C16—H16	0.9300
C3—C8	1.519 (4)	C17—C18	1.388 (5)
C4—C5	1.394 (4)	C17—H17	0.9300
C4—H4	0.9300	C18—H18	0.9300
C5—C6	1.384 (4)	O19—H19A	0.70 (5)
C5—H5	0.9300	O19—H19B	0.75 (4)
C6—C7	1.386 (4)	O20—H20A	0.78 (5)
C6—H6	0.9300	O20—H20B	0.70 (5)
C7—C11	1.513 (4)	O21—H21A	0.91 (6)
C8—O10	1.226 (3)	O21—H21B	0.81 (6)
C8—O9	1.286 (3)		
			
N2—Cu1—N14	166.22 (10)	O10—C8—O9	125.9 (3)
N2—Cu1—O9	80.44 (8)	O10—C8—C3	119.8 (2)
N14—Cu1—O9	100.39 (9)	O9—C8—C3	114.3 (2)
N2—Cu1—O12	79.55 (8)	C8—O9—Cu1	114.62 (16)
N14—Cu1—O12	97.91 (9)	O13—C11—O12	125.8 (2)
O9—Cu1—O12	159.43 (8)	O13—C11—C7	119.7 (2)
N2—Cu1—O19	98.60 (9)	O12—C11—C7	114.5 (2)
N14—Cu1—O19	95.05 (10)	C11—O12—Cu1	114.43 (16)
O9—Cu1—O19	94.79 (9)	C18—N14—N15	105.1 (2)
O12—Cu1—O19	92.87 (9)	C18—N14—Cu1	129.0 (2)
C3—N2—C7	122.1 (2)	N15—N14—Cu1	125.41 (19)
C3—N2—Cu1	118.43 (17)	C16—N15—N14	111.1 (3)
C7—N2—Cu1	119.42 (17)	C16—N15—H15	126 (3)
N2—C3—C4	121.0 (2)	N14—N15—H15	122 (3)
N2—C3—C8	111.8 (2)	N15—C16—C17	108.0 (3)
C4—C3—C8	127.3 (2)	N15—C16—H16	126.0
C3—C4—C5	117.7 (3)	C17—C16—H16	126.0
C3—C4—H4	121.1	C16—C17—C18	105.3 (3)
C5—C4—H4	121.1	C16—C17—H17	127.4
C6—C5—C4	120.6 (3)	C18—C17—H17	127.4
C6—C5—H5	119.7	N14—C18—C17	110.5 (3)
C4—C5—H5	119.7	N14—C18—H18	124.7
C5—C6—C7	118.3 (3)	C17—C18—H18	124.7
C5—C6—H6	120.9	Cu1—O19—H19A	110 (4)
C7—C6—H6	120.9	Cu1—O19—H19B	115 (3)
N2—C7—C6	120.3 (2)	H19A—O19—H19B	101 (5)
N2—C7—C11	111.7 (2)	H20A—O20—H20B	103 (6)
C6—C7—C11	128.0 (2)	H21A—O21—H21B	102 (5)
			
C7—N2—C3—C4	0.4 (4)	C4—C3—C8—O9	173.6 (3)
Cu1—N2—C3—C4	−177.8 (2)	O10—C8—O9—Cu1	−172.0 (2)
C7—N2—C3—C8	−179.6 (2)	C3—C8—O9—Cu1	7.5 (3)
Cu1—N2—C3—C8	2.3 (3)	N2—C7—C11—O13	−172.6 (2)
N2—C3—C4—C5	−0.5 (4)	C6—C7—C11—O13	7.5 (4)
C8—C3—C4—C5	179.4 (3)	N2—C7—C11—O12	6.4 (3)
C3—C4—C5—C6	0.1 (4)	C6—C7—C11—O12	−173.5 (3)
C4—C5—C6—C7	0.5 (4)	O13—C11—O12—Cu1	171.0 (2)
C3—N2—C7—C6	0.2 (4)	C7—C11—O12—Cu1	−7.9 (3)
Cu1—N2—C7—C6	178.3 (2)	C18—N14—N15—C16	−1.2 (4)
C3—N2—C7—C11	−179.7 (2)	Cu1—N14—N15—C16	170.8 (3)
Cu1—N2—C7—C11	−1.6 (3)	N14—N15—C16—C17	1.2 (5)
C5—C6—C7—N2	−0.6 (4)	N15—C16—C17—C18	−0.7 (5)
C5—C6—C7—C11	179.2 (3)	N15—N14—C18—C17	0.7 (4)
N2—C3—C8—O10	173.0 (3)	Cu1—N14—C18—C17	−170.9 (3)
C4—C3—C8—O10	−6.9 (4)	C16—C17—C18—N14	0.0 (5)
N2—C3—C8—O9	−6.5 (3)		

### Hydrogen-bond geometry (Å, º)

**Table:**

*D*—H···*A*	*D*—H	H···*A*	*D*···*A*	*D*—H···*A*
O19—H19*B*···O21	0.75 (4)	2.09 (5)	2.831 (5)	169 (4)
O20—H20*B*···O13	0.70 (5)	2.12 (5)	2.807 (4)	172 (6)
N15—H15···O12^i^	0.93 (4)	1.93 (4)	2.832 (3)	164 (4)
O19—H19*A*···O9^ii^	0.70 (5)	2.12 (5)	2.805 (3)	165 (5)
O20—H20*A*···O10^iii^	0.78 (5)	2.01 (5)	2.784 (4)	171 (5)
O21—H21*A*···O20^iv^	0.91 (6)	2.05 (6)	2.933 (5)	163 (5)
O21—H21*B*···O20^v^	0.81 (6)	2.17 (6)	2.938 (5)	161 (6)

Symmetry codes: (i) *x*+1, *y*, *z*; (ii) *x*−1, *y*, *z*; (iii) *x*−1, *y*+1, *z*; (iv) *x*, *y*−1, *z*; (v) *x*+1, *y*−1, *z*.
